# The Voluntary Hospitals, Patients, Payments and State Aid

**Published:** 1908-04-18

**Authors:** 


### The Hospital

A Journal of

The Medical Sciences and Hospital Administration.

New Series. No. 57. Vol. III. [No. 1129, Vol. XLIV.] SATURDAY, APRIL 18, 1908.

### The Voluntary Hospitals, Patients' Payments and State Aid.

The discussion raised by Mr. Sydney Holland's proposal that where they can afford to do so, hospital patients ought to be asked to pay for the food, medicines, and bandages supplied to them, is one which has always had our support and advocacy. The mover, who we are glad to see carried this resolution at the Hospital Conference, was well justified in making most of the points on which he relied. Dr. Heron's contention that no man who can afford to pay a general practitioner should be admitted as an in-patient to a voluntary hospital is not in accordance with modern hospital experience. It is the out-patient department, not the in-patient section, of many of our hospitals, as at present conducted, which injures the general practitioner. Yet the real remedy for the existing abuses of the latter—the conversion of the out-patient department into an auxiliary to, as opposed to a competitor with, the doctor, by using it for consultation purposes only for suitable cases—found no place in the programme of the late Conference. Why the British Medical Association should have omitted it is a point on which the whole profession desires an explanation.

The mover showed that small payments for food and drugs by those patients who can afford such contributions in cash is not found in practice to be subject to the objections raised against it. So far as the in-patients are concerned, a man may be a fit inmate of a hospital because he cannot procure at home the accommodation and facilities needed for his speedy relief, though he may be able to pay for the cost of his food, drugs, and bandages. This Dr. Heron does not seem to recognise or grasp. Again, there are probably many in-patients who can afford to make such payments, but who could not pay the cost of operations and nursing. Then, too, the supporters of our hospitals are becoming more than ever convinced that there is too much free medical relief given by the hospitals in the present day; and they will soon come to support some such system as that advocated by The Hospital for so many years and now supported by the Hospital Conference. Indeed, the change is urgently called for, and should commend itself to the support of general practitioners, hospital subscribers, and the patients themselves. The last are only too anxious in many cases to have the right to pay all they can for the hospital care which they so urgently need when ill.

We gather from the remarks made by Mr. Holland, and from certain statements which are attributed to him in the *Daily Telegraph*, that the task he has undertaken at the London Hospital is rendering him unduly pessimistic. He states, what is unquestionably not supported by the facts, so far as the experience of the London hospitals during the last twenty years, and especially during the last ten years, is concerned, that "they are in a very serious condition, and that there is no hope for them unless they can call upon the State to help." He repeats the same argument in the *Daily Telegraph*. The truth is that the London hospitals, taking the whole course of their history, were financially never so prosperous as they are to-day. We have not space to give details, but any one interested may study the figures by referring to Burdett's "Hospitals and Charities," 1908. It must suffice for our present purpose to point out that the income of ninety-nine London hospitals in 1896 was approximately £796,000, and that the income of 105 hospitals, including the ninety-nine aforesaid, in 1905 was £1,396,000, an increase in revenue of nearly 100 per cent. in ten years. The case of the London Hospital justifies no such statement as that made by Mr. Sydney Holland, seeing that if it had £30,000 a year more, as we pointed out in a recent article, financially it would be placed upon a basis which, under careful management, should prove sufficient for its purposes for many years to come. There need be no difficulty, as the totals we have quoted prove, in increasing the income of the London to this relatively small extent, if the proper means are adopted to that end. Further than this, all the best informed and most trustworthy hospital opinion is opposed to State aid, in any form, for the voluntary hospitals, because it must mean in the end State control, and the experience of all countries which have tried this system is that such government cripples progress and destroys efficiency in the highest and best meaning of these words. It is always a misfortune when any one who holds a high official position commits himself to statements which do not rest upon the surest foundation. We hope, therefore, the Chairman of the London Hospital will take an early opportunity to put himself right with hospital opinion in regard to State aid for our voluntary hospitals.

